# Injection drug use, unsafe medical injections, and HIV in Africa: a systematic review

**DOI:** 10.1186/1477-7517-6-24

**Published:** 2009-08-28

**Authors:** Savanna R Reid

**Affiliations:** 1 School of Community Health Sciences, University of Nevada at Las Vegas, 4505 Maryland Parkway, Las Vegas, NV 89154, USA

## Abstract

The reuse of injecting equipment in clinical settings is well documented in Africa and appears to play a substantial role in generalized HIV epidemics. The U.S. and the WHO have begun to support large scale injection safety interventions, increased professional education and training programs, and the development and wider dissemination of infection control guidelines. Several African governments have also taken steps to control injecting equipment, including banning syringes that can be reused.

However injection drug use (IDU), of heroin and stimulants, is a growing risk factor for acquiring HIV in the region. IDU is increasingly common among young adults in sub-Saharan Africa and is associated with high risk sex, thus linking IDU to the already well established and concentrated generalized HIV epidemics in the region. Demand reduction programs based on effective substance use education and drug treatment services are very limited, and imprisonment is more common than access to drug treatment services.

Drug policies are still very punitive and there is widespread misunderstanding of and hostility to harm reduction programs e.g. needle exchange programs are almost non-existent in the region. Among injection drug users and among drug treatment patients in Africa, knowledge that needle sharing and syringe reuse transmit HIV is still very limited, in contrast with the more successfully instilled knowledge that HIV is transmitted sexually. These new injection risks will take on increased epidemiological significance over the coming decade and will require much more attention by African nations to the range of effective harm reduction tools now available in Europe, Asia, and North America.

## Introduction

Medical injections performed with used syringes and needles may explain a large part of Africa's intractable AIDS crisis, allowing cyclic transmission within high risk groups treated at sexually transmitted disease clinics, transmitting HIV between closed sexual networks, and infecting individuals who believe they are not at risk [1]. Blood exposures of small volumes resulting from the reuse of unsterile instruments for invasive medical and dental care also carry a meaningful risk of HIV transmission. Significant amounts of viable HIV survive for more than two hours outside the body, whether on sharp surfaces exposed to air or adhering to surfaces within used needles and syringes [2,3]. Under rationing and staffing pressures, this knowledge is often lacking or set aside in sub-Saharan Africa [4].

In South Africa and Ethiopia many health workers consider injections safe when the needle is changed but the syringe is reused, but syringe reuse is practiced even where 97% of health workers recognize single use guidelines [5,6]. South African health workers in public maternity and pediatric wards reused syringes under direct observation in 2005, and 30% of those surveyed did not see the need to use a new needle for each patient [6]. The World Health Organization (WHO) estimates that in 2000 between 17–19% of injections performed in sub-Saharan Africa were administered unsafely [7]. Injection safety has improved in all reporting countries over the last ten years (Benin, Cote d'Ivoire, Ethiopia, Lesotho, Liberia, Malawi, Mali, Rwanda, Swaziland, Tanzania, Uganda and Zimbabwe), but sterilization equipment for other critical items that must be safely reused (e.g., surgical forceps, specula, dental instruments) and appropriate training are lacking in many formal health facilities [8].

Evidence of this role for poverty in the AIDS pandemic has been neglected, if not actively suppressed in HIV epidemiology [9]. Reverse causation has figured prominently in the argument that associations between medical injections and HIV status do not indicate iatrogenic transmission [10]. Yet in all but one of the large cohort studies of HIV incidence that followed HIV negative people in Africa between 1984 and 2006, people who received medical injections were those more likely to acquire HIV. In these studies, the median population attributable fraction (PAF) of HIV incidence associated with receipt of a medical injection was 19% (range 0–54%) [11].

The core public health message that AIDS is transmissible both through sex and through needle reuse has been taught consistently in developed nations because injection drug use (IDU) is common. Many AIDS prevention programs in Africa have set aside injection risks in their communications with the public, perceiving IDU as uncommon. Introducing this information and supporting efficacious infection control in primary health care is vital to protecting patients from HIV as well as other blood borne agents. In addition, a high risk group for blood exposures needs to be acknowledged and targeted for outreach. Africa's growing population of IDU are, in some communities, largely unaware that sharing needles carries a risk of transmitting HIV.

## Injection drug use in Africa

Injection drug use is no longer rare in sub-Saharan Africa. Established along opiate and cocaine transshipment routes up and down both coasts in the 1990s, IDU is now prevalent even among refugees from the interior regions of the Democratic Republic of Congo [12]. The most commonly injected drug in Africa is heroin, followed by cocaine and speedball, a combination of heroin and coke [13]. In 2006 an estimated 0.2% of African adults were using heroin, approaching the global average [14]. In 1997 heroin consumption even exceeded marijuana consumption in Ghana, cutting across all socioeconomic groups and playing a visible role in the domestic economy [15]. Methamphetamine use is also increasing rapidly, and while usually smoked, it is increasingly used in combination with heroin, acting as a gateway drug to more addictive opiates [16].

IDU have been interviewed through treatment centers and the use of snowball sampling (chain referral) in urban Africa and large towns, but the prevalence of IDU in rural Africa has not been assessed. National IDU prevalence estimates from data on urban areas range up to an astounding 1.4% in Mauritius, and prevalence is highest among secondary students, sex workers, and prisoners in Africa. The living situation of male IDU varies from city to city, but most hold only temporary jobs or rely on crime and begging to support their drug habits, and homelessness is common [14,17,18]. In Ghana 48% of IDU are unemployed and involved in petty theft to support their drug habit [15].

Heroin use was introduced in the 1980s in a form called "brown sugar" that is smoked (men call this "chasing the dragon") [19]. Heroin users increasingly adopted IDU when the supply of heroin shifted from the relatively inexpensive "brown sugar" variety to a more refined powder in the 1990s [20]. Injecting is preferred over smoking for the more expensive heroin, as a more efficient high [21].

Most IDU in Africa are male, ranging from 66% in northern Nigeria to 93% in Nairobi, Kenya [17,22]. However many African prostitutes are IDU, and injecting prevalence among female sex workers ranges up to 74% in Mauritius, where one quarter of IDU are sex workers [14,23,24]. Almost all female IDU are sex workers, and female IDU are at greatest risk of HIV infection, with an HIV prevalence two to ten times higher than among male IDU [13,14,25,26]. This reflects both greater exposure through needle sharing and greater exposure through unprotected sex [14,18]. In Dar es Salaam, female IDU report an average of 3 sexual partners per heroin binge, and an average of 61.2 sex partners in the last month [27]. The average is 2.4 partners in a month for men.

Although men and women often inject under different circumstances, injecting practices are readily transferred between them. Tanzanian sex workers share blood with fellow users who cannot afford heroin, in a particularly dangerous practice called "flashblood," which has recently been reported among men as well. One user draws blood back into the syringe after injecting heroin, and passes the syringe to a companion, who then injects the 3–4 mL of blood [28]. This amount of blood carries a high probability of HIV transmission.

**Figure 1:**
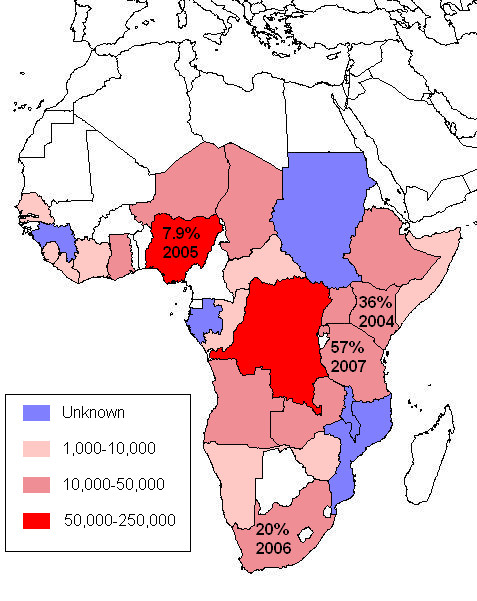
**IDU prevalence and HIV prevalence among IDU in sub-Saharan Africa**.

Patterns of heroin injecting vary from daily or intermittent use for most Nigerian IDU to frequent binging in Dar es Salaam, Tanzania [13,28]. Heroin use now occurs in most large towns in Kenya and Tanzania [29], and is increasing in Cote d'Ivoire, Kenya, Mauritius, Morocco, Nigeria, Egypt, Mozambique, South Africa and Tanzania [30]. IDU is also highly prevalent in Ghana and the Democratic Republic of Congo. Reports are not available for Guinea-Bissau, now considered a narco-state [31]. Figure 1 reports estimated IDU populations in sub-Saharan Africa, and the prevalence of HIV among IDU for the four countries shown where data is available. The latter can be compared with global rates in a review that only reports IDU prevalence for countries also reporting the prevalence of HIV in IDU (Figure three of Mathers et al. (2008)) [32]. Mauritius (not shown) is a small island nation east of Madagascar in the Indian Ocean with an estimated 22,500 IDU. Most estimates are from the UNODC 2008 world drug report [33].

### Risks to Youth

In the town of Malindi in coastal Kenya, heroin use is associated with drug and sex trafficking with European tourists [34]. Here injecting is not considered "cool" among youth ("poa" in Swahili). In contrast, in Tanzania injecting occurs in open-air youth hangouts as well as in more private settings [14]. One in five Tanzanian youth surveyed in 1991 had ever tried heroin, and drug overdose is the most common method of parasuicide in young adults [35].

IDU is increasing among youth in most parts of Africa, and has the potential to accelerate HIV transmission in the very demographic with the highest HIV incidence, as sexually active IDU may bridge concentrated and generalized epidemics among young adults. Injecting behavior in youth is associated with ease of access to heroin and unemployment [36,37]. In a large sample of IDU in Dar es Salaam, 76% of males lived with their parents at the time of the interview, as did 21% of female IDU [28]. For street children injecting is common and may be especially dangerous. In a small sample of street children in the Great Lakes region (in East Africa), 43.5% reported sharing syringes or other instruments when using drugs [38].

In South Africa the average age at onset of heroin use is 20 [24]. One third of IDU in Kenya and Tanzania are under age 25, compared to only 2% of IDU in Nigeria, although in Nigeria, a relatively large proportion of tertiary students had ever injected heroin (2.4%) and student heroin use dates back to the 1980s [17,28,39]. In Mauritius injecting is even more prevalent among students at 4.3% [14]. Ethiopian youth, by contrast, are no more likely than low-risk groups to have ever injected drugs [23].

### Crime and Prisons

Drug criminalization and drug-related crime contribute to high IDU prevalence in African prisons. Injecting has been reported in prisons in Cote d'Ivoire, Mauritius and Ghana. From a human rights perspective, the threat of HIV and hepatitis C transmission in prison warrants harm reduction interventions such as providing needles for IDU, and this would also mitigate the role of prisons as disease reservoirs in the community [40].

Drug treatment and HIV counseling could reach a large fraction of IDU through prisons, as criminalization has driven IDU underground and made them a hard to reach population on the street. In Mauritius 17% of juvenile offenders and 50% of adult offenders are IDU, and an estimated 16% of IDU were imprisoned at some time in 2005 [14,19]. In Ghana in 2007, more than a third of prison inmates had ever injected drugs, even though only 10% had been arrested for drug trade or possession [41,42]. In South Africa, only 1.3% of arrestees are IDU, but 17% of IDU have been arrested in a year [43].

In Europe, Iran, Australia and Russia, harm reduction programs for prisons address the great HIV transmission risk injecting in prison entails [44]. Similar efforts would be appropriate in much of Africa, considering that injecting dominates HIV transmission for inmates in drug transshipment countries. In South Africa 45% of IDU in prison are HIV positive, compared to 22% of other arrestees [24]. Among inmates in Ghana, injection drug use carries an odds ratio of 5.7 for HIV (95% CI 2.4–12.8), making this the strongest behavioral risk factor for HIV infection while in prison [41]. In Cote d'Ivoire 7% of all prisoners have shared needles while in prison, and among IDU imprisoned in Ghana and Mauritius, 72% and 31% had ever shared needles [14,41,45].

### Needle Sharing

Knowledge that a clinically significant amount of viable HIV can survive outside the body on blood-contaminated instruments for several hours is not widespread in Africa [2,3,46]. Table 1 reports the rate of needle sharing in IDU in Africa. A large proportion of IDU regularly share syringes, and only 25% of Nigerian IDU report knowing that doing so carries a risk of HIV transmission. The HIV transmission risk is known to more IDU in Kenya (73% in Nairobi) and virtually all secondary students in Mauritius (98.5%) [14,47]. Yet group needle ownership is common among IDU on the Kenyan coast, and among IDU who know they are HIV positive in Kenya up to 27.6% reported passing their needle to someone else in the past year [14,34]. Needle sharing with sex partners is particularly common among female IDU [48]. In Mombassa users typically use the same needle for 1–3 days, and those who store a syringe (usually at or near an injecting gallery) report it must be hidden, as another user will often steal it [34].

**Table 1:** Rates of needle sharing reported by IDU in five sub-Saharan African countries

**Country**	**Population**	**Needle sharing**	**Ref**.
Kenya	IDUs	27–81% (ever)	[14]

Kenya	Injecting heroin users in Nairobi	28–52% (past 6 months)	[14,34]

Kenya	Female IDUs in Nairobi	28–44% (no timeframe)	[48]

Mauritius	IDUs	80% (past 3 months)	[14]

Mauritius	Injecting sex workers	77% (ever)	[14]

Nigeria	Injecting heroin users	11–15% (past 6 months)	[14]

South Africa	Injecting heroin users in Cape Town	56–86%% (past 30 days)	[14,24,43]

Tanzania	Female IDUs in Dar es Salaam	6% (no timeframe)	[29]

Tanzania	IDUs in Zanzibar	46% (ever)	[49]

IDUs – injection drug users

The syringes available to drug users in East Africa are large gauge and typically damage small veins early in the injection careers of heroin users [30]. Reuse rapidly blunts the needles. Larger gauge and blunted needles transmit larger volumes of blood and likely pose a greater HIV transmission risk when shared.

The HIV prevalence among IDU who share needles is high, reaching 28% in Zanzibar (vs. 5% in IDU who do not) [49]. The IDU population in Kenya is believed to be in decline primarily because of HIV-related mortality [34]. In Mauritius, HIV prevalence among drug users has come to dominate the AIDS epidemic over the course of only a few years, so that 92% of new HIV infections in 2005 were identified in IDU [14]. In Kenya, for comparison, only 4.8% of new HIV infections are attributed to IDU, although the HIV prevalence among IDU is five times greater than in the general population [21].

In Dar es Salaam the HIV prevalence among IDU varied from 0–90% across neighborhoods in 2006, averaging 57%. This variation was notably unrelated to religion (neighborhoods with fewer or more Islamic families) or socioeconomic status; both highest and lowest prevalence neighborhoods were culturally mixed [25]. Here and particularly in Zanzibar, the perception that Muslim communities are not at risk from HIV for cultural reasons, and a particular reluctance to acknowledge culturally unacceptable sexual behavior and injection drug use, may pose a special challenge for harm reduction efforts. Effective precedents in harm reduction for Muslim communities in Uganda and Senegal demonstrate that these taboos are not an intractable obstacle to AIDS prevention programs [50].

### Drug Policy and Services

In most African countries resources for harm reduction are still lacking and drug use is marginalized as a crime [14]. Recent regional cooperation has led to the creation of a data base on African NGOs active in demand reduction activities, but overall OAU activities reflect a political preference to focus on controlling drug supply [51]. National and regional drug policy goes beyond criminalization in only a few instances, and international conventions are contradictory, often curbing resources for harm reduction on the grounds that they condone drug abuse, plainly under pressure from the U.S. [52].

Table 2 presents the most recent IDU prevalence estimates (among adults), and identifies existing harm reduction policies and non-governmental organizations in countries with reported injection drug use [33]. In 2004 in psychiatric hospitals, 33%, 8% and 30% of patients in Mozambique, Zambia, and Tanzania respectively presented for heroin addiction treatment [53]. Drug treatment demand has been met only for those who can pay, except for the services of only a handful of non-governmental organizations, and to redress this inequity public funds for drug treatment are increasingly being shifted back to primary health care [13]. Injection drug users are reluctant to present for public services, however, fearing they will be turned over to the authorities [54-56].

**Table 2:** IDU prevalence in 2008 and harm reduction resources in sub-Saharan Africa [33,55,56]

**Country**	**IDU prevalence**	**Harm reduction NGOs and government programs**
Angola	0.18%	NGO(s) involved in rehabilitation

CAR	0.09%	NGO(s) involved in rehabilitation

Congo	0.08%	Mental health policy includes rehabilitation

Cote d'Ivoire	0.08%	Mental health policy includes rehabilitation

		NGO(s) involved in rehabilitation

DRC	0.6%	Mental health policy includes rehabilitation

		NGO(s) involved in rehabilitation

Ethiopia	0.08%	None identified.

Ghana	0.05%	Mental health policy includes rehabilitation

Kenya	0.18%	NGO: Omari Project

		Government programs include opioid substitution

Liberia	0.2%	NGO: Student Aid Liberia Inc.

Mauritius	1.8%	NGO: Prevention Information et Lutte contre le Sida

		Government programs include needle exchange and methadone treatment

Mozambique	Unknown	Government programs include drug treatment at psychiatric hospitals

		NGO(s) involved in rehabilitation

Namibia	0.08%	NGO(s) involved in rehabilitation

Niger	0.09%	NGO(s) involved in rehabilitation

Nigeria	0.35%	NGO: Nigerian Friends for Harm Reduction

Senegal	0.08%	Mental health policy includes rehabilitation

		NGO(s) involved in rehabilitation

Sierra Leone	0.03%	NGO(s) involved in rehabilitation

Somalia	0.09%	NGO(s) involved in rehabilitation

South Africa	0.15%	Government programs include opioid substitution and demand reduction

		NGO(s) involved in rehabilitation

		NGO: RAVE Safe

Tanzania	0.09%	Government programs include counseling and rehabilitation

		NGO(s) involved in rehabilitation

		PEPFAR/USAID providing referral for voluntary HIV counseling and testing and for drug treatment

Uganda	0.1%	Mental health policy includes rehabilitation

		NGO(s) involved in rehabilitation

Zambia	0.18%	Government programs include drug treatment at psychiatric hospitals

		NGO(s) involved in rehabilitation

Zimbabwe	0.09%	NGO(s) involved in rehabilitation

CAR – Central African Republic, DRC – Democratic Republic of Congo (formerly Zaire), IDU – injection drug use

In Tanzania, drug policing is highly visible, but demand reduction has not received the same attention, and injection drug use has been driven underground [57]. Some 30 heroin addicts are received for emergency psychiatric services in Muhimbili Medical Center in Dar es Salaam every year [58]. In 2004 both the President's Emergency Plan for AIDS Relief (PEPFAR) and USAID backed a community based outreach program to reach IDU in Tanzania and refer them to voluntary counseling and testing (VCT) and HIV and drug treatment [57]. Addiction services are available from NGOs and mental health and family counseling, and in psychiatric agricultural rehabilitation villages [55,57]. These villages, developed in 1969, provide occupational therapy as well as mental health services, and importantly they are also self-supporting. Federal support for public health services has contracted dramatically under structural adjustment policies, undermining both primary health care safety and services for drug treatment.

In coastal Kenya a small service for heroin addicts, the Omari Project, has incorporated injection safety into its counseling sessions [34]. However access to addiction services in Kenya is limited primarily to residential facilities serving males who can pay for care [59]. Most drug treatment in Kenya goes on at government hospitals instead.

In South Africa drug treatment has been accessible mostly to white IDU who can make co-payment for clinical services [14,60]. Public funding for drug treatment is being scaled back and integrated into primary health care networks to redress this inequality, as IDU prevalence increases among colored and black South Africans [14]. Here demand reduction activities have focused on at-risk women, and on youth (e.g., the "Ke Moja – No thanks, I'm fine!" drug awareness campaign, and a classroom-based leisure, life-skill and sexuality education curriculum, "HealthWise") [60]. Allowing IDU access to new injecting equipment is not promoted, however. In South Africa 48% of IDU reported having been denied needles within the last year at a hospital or pharmacy [14].

Mauritius' 2006 HIV and AIDS Act established Africa's first needle exchange and methadone maintenance program [57]. This reaction to explosive HIV transmission among IDU in an otherwise low-prevalence population may not be duplicated in countries with greater HIV prevalence. Through early 2009, there are no other needle exchange programs in sub-Saharan Africa [61]. However, in 2007 the Sub-Saharan African Harm Reduction Network (SAHRN) was formed, and NGOs, researchers and UN representatives from eleven African countries met to discuss drug harms and policies [62].

## Medical injections and HIV in Africa

Estimates of the relative importance of unsafe medical injections in the AIDS pandemic vary across orders of magnitude. This is because the probability an individual unsafe medical injection will transmit HIV is not known, and estimates supported in the peer reviewed literature range from 0.1% to 6.9%. These estimates are drawn from four types of empirical evidence: (1) rates of HIV infection from needle-stick injuries (any accidental scratch or jab commonly injuring a health worker while administering an injection to an HIV infected patient) [63]; (2) HIV incidence among IDU who share needles [63-65]; (3) retrospective analysis of large iatrogenic HIV outbreaks [66]; and (4) laboratory examinations of used syringes collected in the field [67,68]. Although interpretation of the available evidence is divided, these four types of estimates of the probability a medical injection will transmit HIV all include the range from 1.9–2.3%. The WHO models the probability of transmitting HIV as 1.2% [69].

HIV prevalence is stabilizing in much of sub-Saharan Africa, but the AIDS burden on health care is still increasing as more patients progress to advanced HIV disease, unfortunately outpacing the availability of antiretroviral drugs. Updating the WHO's model of the global burden of disease from unsafe injections (describing the epidemic in 2000), to account for the elevated clinical prevalence of HIV, an estimated 12–17% of new HIV infections in 2007 could be attributed to unsafe medical injections alone [70]. Hospital acquired infections from other invasive procedures have not been estimated, but assisted delivery has been linked to excess HIV infections across Africa and visible blood has been observed on arterial forceps, sutures and other equipment that contacts patients in maternity and pediatric wards [71,7].

Hundreds of recorded cases of HIV positive children with HIV negative mothers indicate that the harm to children has been substantial [72]. Today most African countries use only auto-disable (self-destructing, non-reusable) syringes for immunizations, but other risks to children that persist include invasive procedures, dental care, and non-immunization injections. In South Africa auto-disable syringes are not required for immunizations, and the HIV prevalence in children is too high to be explained by mother-to-child transmission alone [73]. Moreover the incidence of HIV in children no longer breastfeeding and already immunized (ages 2–14) is 0.5% per year in South Africa [74].

The WHO's model of injection risks in the year 2000 estimates that African adults receive on average 2.1 injections per year, and that almost one in five injections are unsafe [69]. More recent data on unsafe injection frequency, available from 12 of the 14 countries in Table 3, demonstrate significant improvement. The probability that an adult will receive an unsafe medical injection in a year varies from 0.1% to 22% (lowest and highest in Lesotho and Rwanda), but the median is only 4.4% [8]. In these countries unsafe injection risks are generally greater for men, for the poor, and in rural areas [8].

**Table 3:** Unsafe injection frequency and sterilization equipment coverage in sub-Saharan Africa 2002–2007

**Country, survey year**	**Unsafe injections per person in past year**	**Clinics with sterilization equipment (%)**
Benin, 2006	4.2	No information

Cote d'Ivoire, 2005	5.3	No information

Ethiopia, 2005	7.6	No information

Ghana, 2002	No information	67

Kenya, 2004	No information	60

Lesotho, 2004	0.1	No information

Liberia, 2007	7.8	No information

Malawi, 2004	3.9	No information

Mali, 2006	1.7	No information

Rwanda, 2005/2007	22.0	83

Swaziland, 2006	4.6	No information

Tanzania, 2005/2007	3.3	65

Uganda, 2005/2007	5.3	68

Zimbabwe, 2006	3.3	No information

More than a third of the population of sub-Saharan Africa (living in Nigeria, Uganda, Malawi, the Democratic Republic of Congo, and Burkina Faso) should be at much lesser risk of unsafe injections, as they are protected by national bans on the use of disposable syringes that can be unsafely reused. Other injection safety interventions have been funded under the President's Emergency Plan for AIDS Relief (PEPFAR) through Making Medical Injections Safer projects. These interventions and those of the WHO's Safe Injection Global Network (SIGN) have reduced the frequency of unnecessary injections, reduced the risks posed by improper disposal of sharp medical waste, and produced and disseminated infection control guidelines to improve clinical practice. However these programs ignore larger problems with infection control capacity in African health care settings, as reported in Table 3[8].

AIDS researchers and health workers under rationing pressures face a conflict of interest in acknowledging and investigating risks to transmit HIV from patient to patient, as this may undermine public confidence in the competence and motivation of researchers and health workers, leading to under-utilization of essential health services and to preventable morbidity and mortality [75]. Ministries of Health have a duty to resolve this ethical dilemma while scaling up primary health care services. Informing patients and health workers of the seriousness of HIV transmission risks in minor blood exposures and equipping the health care system to cope with the full demands of infection control will be necessary to avert further iatrogenic HIV transmission. These responsibilities go beyond injection safety interventions such as using only auto-disable (self-destructing, non-reusable) syringes.

WHO assurances that medical injection risks are minimal are not credible, and reflect a pattern of suppressing evidence that heterosexual sex explains less than 90% of HIV transmission in Africa [1]. Where evidence of harm is egregious, leading AIDS researchers have invoked a relativistic standard, characterizing a 1% prevalence of HIV positive children with HIV negative mothers (in six major African cities) as representing a "low" risk of patient-to-patient HIV transmission [76]. In a crude irony concerning the social construction of disease, the WHO is defending a 90% estimate that was arrived at by a process of elimination; that is, not on the basis of positive evidence that 90% of HIV infections can be traced to sex in Africa [77]. In fact infection tracing has been consistently avoided in cases of reportedly non-sexual HIV transmission identified in epidemiological research. Self-reported virgins with HIV, and research subjects with incident infections who claim not to have had sex over the study interval, have been classified as evincing "social desirability bias," by denying epidemiologically implicit sexual behavior.

Blood exposures were of interest to HIV epidemiologists in the 1980s, before a consensus focusing on heterosexual transmission was established for Africa, but even transfusion risks were considered intractable at an early stage. Early Western experts' statements concerning the place of infection control in HIV prevention efforts in Africa were highly pessimistic [1]. For example, "one cannot hope to prevent reuse of disposable injection equipment when many hospital budgets are insufficient for the purchase of antibiotics." This statement appears in an important 1986 article whose authors include the heads of WHO's Global Programme on AIDS and later UNAIDS for most of the next 21 years [77]. The problem has not worked itself out, and cannot wait for the day when rationing does not limit the options of health workers in sub-Saharan Africa.

## Conclusion

Injection drug use has increased rapidly during the recent past throughout sub-Saharan Africa, with the greatest increase in Mauritius, and the greatest numbers of IDU in West-Central Africa. Projecting a similar rate of increase through the year 2015, IDU prevalence could reach 0.24% in Southern Africa, 0.08% in East Africa, and 0.19% in West-Central Africa. For comparison, in the U.S. the prevalence of heroin use (primarily administered by injecting) has stabilized at around 0.2%,^78,33 ^and the prevalence of methamphetamine injecting has risen to 0.3% of adults under 50 [33,78,79]. Although IDU prevalence is greatest and expanding most quickly in major drug transshipment countries, habitual injecting has penetrated far beyond the periphery of major ports and airports, observed even among refugees from the interior of the Democratic Republic of Congo.

HIV prevalence among IDU can also be expected to increase, as the scant drug treatment and harm reduction activities in sub-Saharan Africa are unlikely to impact upward trends that have been documented in Nigeria and South Africa. Interventions to raise awareness of the HIV transmission risk from sharing needles are needed, particularly in Nigeria. Outreach (1) to out-of-school youth as well as students, (2) to female sex workers' clients as well as at-risk women, and (3) to unemployed adults and the homeless, as well as IDU who can afford residential treatment, will be needed. Support for harm reduction spending may hinge on recognition that concentrated HIV epidemics among IDU are relevant to the spread of HIV among sexually active young adults in Africa's generalized epidemics.

For the protection of patients, accurate information that HIV can survive outside the body in blood-contaminated instruments and on sharps must be taught, and suspected iatrogenic HIV cases should be traced through the implicated clinics and investigated to identify and prevent other cases. These efforts will in no way detract from the message that HIV is sexually transmitted, even if it is evident that sexual transmission explains less than 90% of infections in Africa. Public awareness of HIV transmission risk from other prevalent skin-piercing procedures (such as tattooing, shaving with an unsterilized razor, or unsterile dental care) is also poor in Africa, and should be addressed simultaneously [46,80,81]. Introducing this information and supporting effective infection control in primary health care could significantly reduce HIV transmission in Africa.

## Competing interests

The author declares that they have no competing interests.

## Authors' contributions

SR carried out the literature search, reviewed the studies identified by search, created the text, and created the illustration. SR is the sole author. All authors read and approved the final manuscript.
